# Assessing Somatosensory Utilization during Unipedal Postural Control

**DOI:** 10.3389/fnsys.2017.00021

**Published:** 2017-04-11

**Authors:** Rahul Goel, Yiri E. De Dios, Nichole E. Gadd, Erin E. Caldwell, Brian T. Peters, Millard F. Reschke, Jacob J. Bloomberg, Lars I. E. Oddsson, Ajitkumar P. Mulavara

**Affiliations:** ^1^Department of Health and Human Performance, University of HoustonHouston, TX, USA; ^2^KBRwyleHouston, TX, USA; ^3^Neuroscience Laboratory, NASA Johnson Space CenterHouston, TX, USA; ^4^Department of Physical Medicine and Rehabilitation, Program in Rehabilitation Science, University of MinnesotaMinneapolis, MN, USA; ^5^Recaniti School for Community Health Professions, Ben-Gurion University of the NegevBeersheba, Israel

**Keywords:** balance control, unipedal stance, somatosensation, sensory biases, stabilogram-diffusion analysis

## Abstract

Multisensory—visual, vestibular and somatosensory information is integrated for appropriate postural control. The primary goal of this study was to assess somatosensory utilization during a functional motor task of unipedal postural control, in normal healthy adults. Assessing individual bias in the utilization of individual sensory contributions during postural control may help customization of rehabilitation protocols. In this study, a test paradigm of unipedal stance control in supine orientation with and without vision was assessed. Postural control in this test paradigm was hypothesized to utilize predominantly contributions of somatosensory information from the feet and ankle joint, with minimal vestibular input. Fourteen healthy subjects “stood” supine on their dominant leg while strapped to a backpack frame that was freely moving on air-bearings, to remove available otolith tilt cues with respect to gravity that influences postural control when standing upright. The backpack was attached through a cable to a pneumatic cylinder that provided a gravity-like load. Subjects performed three trials each with Eyes-open (EO) and Eyes-closed (EC) while loaded with 60% body weight. There was no difference in unipedal stance time (UST) across the two conditions with EC condition challenging the postural control system greater than the EO condition. Stabilogram-diffusion analysis (SDA) indicated that the critical mean square displacement was significantly different between the two conditions. Vestibular cues, both in terms of magnitude and the duration for which relevant information was available for postural control in this test paradigm, were minimized. These results support our hypothesis that maintaining unipedal stance in supine orientation without vision, minimizes vestibular contribution and thus predominantly utilizes somatosensory information for postural control.

## Introduction

Postural control is one of the most fundamental motor tasks in which we integrate input from the visual, vestibular and somatosensory systems (Horak and Macpherson, 1996; Peterka, 2002). Healthy individuals and special populations are found to have sensory utilization preferences under certain conditions for postural control tasks. The degree to which sensory inputs are weighted and reorganized in discordant conditions varies by individual subjects (Streepey et al., 2007; Isableu et al., 2010). Also, individuals’ innate sensory weighting can inform their ability to adapt to a discordant sensory environment. For example, subjects who rely more on vision for control of movement have more difficulty adapting their walking and postural control strategies in new sensorimotor environments. This result indicates that visual dependency may predict decreased ability to adapt to novel environments (Brady et al., 2009, 2012; Hodgson et al., 2010; Eikema et al., 2013). Thus, tests that delineate individual differences in visual, vestibular or somatosensory bias while performing a sensorimotor task may serve as predictors of sensorimotor adaptability in discordant conditions.

Visual utilization can be tested independently using the Rod and Frame Test (Isableu et al., 2010), Embedded Figures Test (Witkin, 1971), or the Treadmill Visual Dependency Test (Brady et al., 2012). Vestibular utilization during postural control can be evaluated by dynamic head pitch tilts while trying to balance on a sway-referenced support surface with Eyes-closed (EC; Paloski et al., 2006) using the Neurocom Balance Manager (Natus Medical Incorporated, Pleasanton, CA, USA). However, there is no test protocol to directly assess the unique contributions of somatosensory information for postural control. This is because various sensory receptors contribute towards somatosensation, such as the muscle spindles, joint receptors, Golgi tendon organs, foot pressure and mechanoreceptors in the skin (Meyer et al., 2004). A typical way to assess the contribution of some of these somatosensory cues during upright stance is by using an intervention like vibration (Kavounoudias et al., 2001; Dettmer et al., 2013; Temple et al., 2016), anesthetization of the foot through restriction of blood supply to the foot (Horak et al., 1990), or injection of an anesthetic (Meyer et al., 2004). However, intervention protocols can only selectively perturb one or two sets of sensory receptors at a time (e.g., bottom of feet or ankle or muscles), but not all. Further, these anesthetization protocols help assess the importance of specific receptor inputs during postural control by excluding their contributions in the presence of other sensory modalities that may compensate for their absence. Additionally, external perturbations of a sensory system (e.g., vibration stimulation of muscle tendon) may trigger specific or transient motor programs that may not be related to a continuous regulation of balance. Thus, there is no test protocol to directly assess the unique contribution of all the somatosensory cues from the feet and lower body towards perception and control of body position during quiet stance without or by minimizing the influence of any graviceptors (vestibular and non-vestibular) and without any external intervention.

The primary goal of this study was to assess somatosensory utilization during a functional motor task of unipedal postural control, in normal healthy adults. This was accomplished using a test paradigm of unipedal stance in supine orientation with and without vision. In upright standing postural control tasks, the orientation of the body relative to gravity can provide fundamental information to organize the necessary multisensory transformations for motor control (Wiener and Berthoz, 1993). In our test paradigm, the gravity vector was perpendicular to the orientation in which subjects were required to perform the posture task. The supine posture in our test paradigm thus ensured that information of orientation of the body relative to gravity was not useful and did not contribute to postural control. Hence, postural control in this testing paradigm was hypothesized to utilize predominantly contributions of somatosensory information from the feet and ankle joint, with minimal vestibular input.

## Materials and Methods

### Subjects

Fourteen subjects (10 male, 4 female) were recruited from the Human Test Subject Facility at NASA-Johnson Space Center (JSC) in Houston, TX, USA. All subjects had passed the equivalent of an Air Force Class III physical examination within 12 months of beginning the study. No subject had any reported history of otologic, neurologic, cardiovascular, orthopedic, or traumatic disorders. For the 14 subjects, mean age was 41.4 ± 7.8 years (mean ± standard deviation), mean height was 175.3 ± 11.6 cm and mean weight was 81.9 ± 17.2 kg, and all subjects had normal or corrected-to-normal vision. The study was performed according to the Declaration of Helsinki. The experimental protocols were approved by the Institutional Review Board at NASA-JSC. All subjects gave their written informed consent before participating in the study and were free to withdraw at any time. The age of our subjects was higher than usually seen in studies with young, healthy individuals as our subject pool consisted of age-matched controls for astronauts who are typically between 38–50 years of age at the time of their spaceflight.

### Experimental Protocol

**Figure 1:**
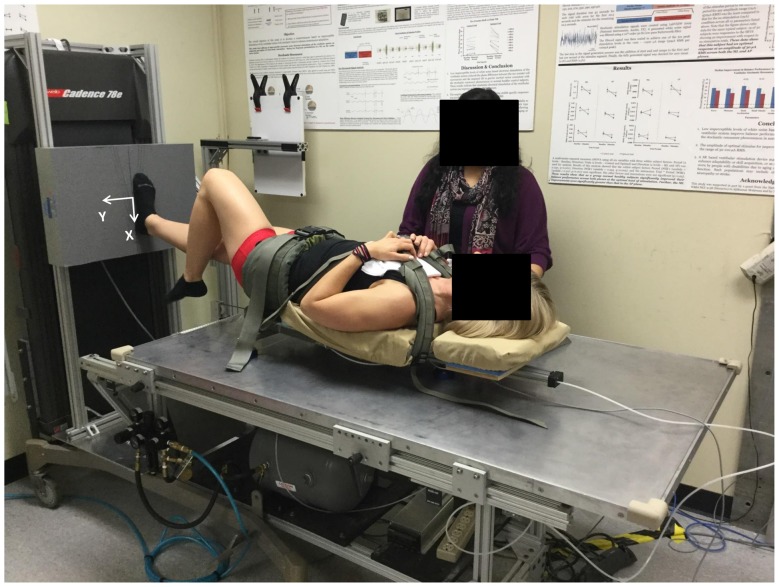
**Subject performing unipedal stance trial on the Gravity-Bed.** The coordinate system used is shown marked on the force plate.

Postural control was evaluated using a unipedal task on the Gravity-Bed (Figure 1; Oddsson et al., 2007, 2015). Subjects “stood” on one leg in a supine position while strapped to a backpack frame fitted with a friction-free device having air-bearings at its interface with the smooth flat table surface that allowed the subject to move freely in the frontal plane. The backpack frame was attached to a pneumatic cylinder that provided different levels of a gravity-like force along the subject’s superior/inferior axis that the subject must balance against to remain “upright”. The attachment point was positioned to be near the center-of-mass (COM) of the subject when lying supine and strapped to the backpack frame. A pelvic-belt and two cross-shoulder straps were used to secure the subject to the frame and distribute the load evenly. Subjects carried out three trials each with Eyes-open (EO) alternated with EC while loaded with 60% of their body weight. There was a recovery period of a minimum of 30 s or a period as long as requested by the subjects between successive trials, in which subjects used both feet to maintain balance and kept their EO. From a bipedal stance in the supine position, subjects were instructed to raise one leg, and find a stable unipedal postural stance. The trial commenced after the subject verbally said “Ready” for EO trials, or “Eyes-closed” for EC trials and continued for 45 s or till the time of fall, whichever occurred first. Sixty percent of body weight was chosen after several subjects were tested in a pilot study before the implementation of this study protocol to assess the comfort levels of subjects from 20% to 100% body weight while performing this task on the Gravity-Bed device. Subjects were allowed to choose where they wanted to keep their raised leg (e.g., how much to flex at knee or hip, without touching the weight-bearing leg), but with the constraint that they had to maintain that position for the entire duration of each of the six individual trials. Subjects wore socks and “stood” on their self-selected dominant leg (all subjects except one used their right foot for weight bearing in the unipedal stance), with their foot externally rotated by 10° about the ankle joint, arms-crossed across the chest. Subjects were instructed to: “use your sense of sway about the ankle and pressure changes under the foot to maintain balance and stand as still as you can”. Subjects were given a minimum of 2–3 practice trials while standing on both feet and swaying about their ankle joints without bending at the knee or the hip to get familiarized with balancing against the load while supine. The maximum length of each trial was 45 s.

Subjects “stood” on a force plate (Kistler 9286B, Kistler, Amherst, NY, USA) that recorded reaction forces and moments during the trial. An inertial measurement unit (IMU) motion sensor (MTx, Xsens North America Inc., Los Angeles, CA, USA) attached to the backpack frame near the COM of the subject recorded upper body postural responses. A single data acquisition program (LabVIEW, National Instruments, Austin, TX, USA) collected time-synchronized data from the force plate and the IMU. The force plate data were filtered using an anti-aliasing filter implemented in the force plate signal-processing hardware with cut-off frequency at 200 Hz before being sampled at 1000 Hz. The IMU data were sampled at 100 Hz. A trial was classified as a “fall” if the subject moved their raised foot (e.g., moved it toward or away from the weight-bearing foot or touched the force plate), spread their arms (i.e., uncrossed them), moved the weight-bearing foot to maintain balance (e.g., rotated the foot on the force plate from the starting position), or opened their eyes during EC trials (Springer et al., 2007). A trial was considered “successful” if the subject maintained “balance” for at least 25 s. If a fall happened within 25 s duration, the trial was repeated following a recovery period of 30 s. If repetition was required, the maximal number of trials was limited to eight.

### Data Analysis

**Figure 2:**
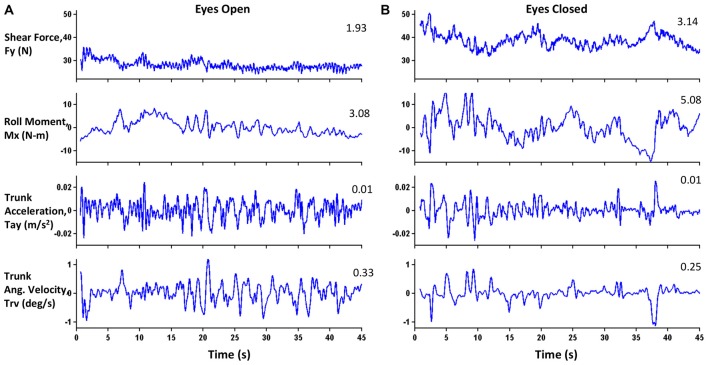
**Exemplar plots of four parameters for one subject under (A)** Eyes-open (EO) and **(B)** Eyes-closed (EC) conditions. Numerals in the top right of each panel represent RMS values. For this subject, the RMS values for the EC condition were greater than or equal to those for the EO condition for all parameters except Trunk Angular Velocity (Trv).

All data analyses were performed using in-house scripts and functions programmed in MATLAB (MathWorks, Natick, MA, USA). The force plate and IMU data were filtered at 10 Hz using a first-order, zero-phase response, low-pass Butterworth filter. Figure 1 also shows the coordinate system used during the data collection. The origin of the force plate coordinate system was located at the center of the force plate. The *Y* axis was an Earth-horizontal axis, and its positive direction was towards the “left” side of the subject. The *X* axis was an Earth-vertical axis, and its positive direction was toward the floor. On the Gravity-Bed, subjects could only move in their medio-lateral (ML) direction (*Y*-direction) with respect to the table surface. For trials in which a fall happened after 25 s, data after the onset of the fall were removed to allow calculation of various parameters. The onset of a fall was detected as the point where center-of-pressure (COP) velocity was 5% of the maximum COP velocity in the ML direction assessed going backward from the end of the trial. Trials with falls were marked to have ended when the backpack frame touched the edge of the table. The time duration until the balance was lost, termed unipedal stance time (UST), was measured. Figure 2 shows example plots of the four parameters (shear force (Fy), roll moment (Mx), linear acceleration of the trunk (Tay), and roll angular velocity of the trunk (Trv)) for one subject during the posture task for both EO and EC conditions. Shear forces are exerted against the support surface whenever the body’s COM accelerates in the ML direction. Horak and Nashner (1986) and Horak et al. (1990) have extensively studied the peak shear force observed in healthy subjects who primarily used a “hip” strategy and found an increase in both hip motion and horizontal shear force. Peak-to-peak amplitude of Fy (ML shear force) was compared with a theoretical maximum shear of 25 pounds (11.4 kg; Horak et al., 1990; Mirka and Black, 1990; Nashner and Peters, 1990; Di Fabio and Foudriat, 1996; Neurocom, 2009) to obtain a strategy score, which assessed the relative use of movement about the ankle, hips and upper body to maintain balance during the trials.

**Figure 3:**
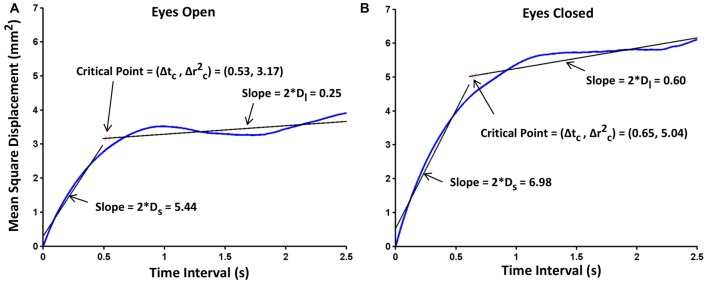
**Exemplar stabilogram-diffusion plots for one subject, under (A)** EO and **(B)** EC conditions. The straight lines in black, fitted to the short- and long-term regions are used to estimate the stabilogram-diffusion parameters. Diffusion coefficients, critical mean square displacements and critical time intervals are also shown on the plots. Hurst exponentials were obtained by corresponding log-transformations of these plots.

The postural performance was analyzed using stabilogram-diffusion analysis (SDA; Collins and De Luca, 1993), which models COP trajectory as a series of correlated random walks. It is a common technique used to address the dynamic nature of the COP motion. Numerous studies have used measures from SDA characterizing the underlying postural control process (Collins and De Luca, 1993; Chow and Collins, 1995; Collins et al., 1995; Mitchell et al., 1995; Meyer et al., 2004; Hernandez et al., 2016). SDA provides several measures that quantify the stochastic behavior of the COP profile and these measures are intended to provide information on the underlying control processes at work during quiet standing. Stabilogram-diffusion plots were constructed, which show mean square displacement of the COP in the ML direction vs. time interval (linear vs. linear and log vs. log plots). They were constructed for each subject by averaging the three trials for each condition. Diffusion coefficients (D) were obtained from the slopes of these linear plots. They represent an average measure of the stochastic activity of the random walker and can be thought of as an indicator of the relative stability of the system. A larger D implies less tightly regulated or “more random” underlying control process driven by higher frequency and/or amplitude of the COP. Figure 3 show exemplar stabilogram-diffusion plots for EO and EC conditions for a representative subject. Scaling exponents (H) were obtained from the slope of the log-log version of the stabilogram-diffusion plot. A value of H equal to 0.5 would indicate a perfectly random walk with equal chance of reversing sway or continuing in the same ongoing direction. For values of H greater than 0.5, the COP displays *persistence*, a behavior that is indicative of a predominance of open-loop control wherein there is a tendency to continue sway in an ongoing direction and move away from some relative equilibrium point. For values of H less than 0.5, the COP displays *anti-persistence*, a behavior indicating a predominance of closed-loop control wherein there is a tendency to reverse sway direction and return to a relative equilibrium point, i.e., past and future sway increments are negatively correlated (Collins and De Luca, 1993). These coefficients were estimated for both short-term (D_s_ and H_s_) and long-term (D_l_ and H_l_) regions which are representative of open-(short-term) and closed-(long-term) loop control characteristics, respectively. Critical time interval (Δ*t_c_*) and critical mean square displacement (Δ$r_{\text{c}}^{2}$) were also estimated from the linear plots. These are defined as abscissa and ordinate, respectively, of the critical point—the intersection of the least squared error fit lines over the short- and long-term regions. The critical point estimates the transition from predominantly open- to predominantly closed-loop control. Consequently, since subjects were instructed to stand as still as possible, the critical mean square displacement would indicate the perceptual COP sway threshold where subjects, on average, initiated balance control. The transition from the long-term region to the saturation region occurs at shorter time intervals in unipedal stance than in bipedal (Chow and Collins, 1995), thus, the SDA was carried out only up to a maximum time interval of 2.5 s rather than the 10 s that is typically used for bipedal stance (Collins and De Luca, 1993; Meyer et al., 2004). The stabilogram-diffusion plots were calculated using 45,000 data points for each trial (45 s long trial sampled at 1000 Hz unless it was a partial trial) and then averaged over three trials. There were 1–2 partial trials for five subjects. These partial trials had data for at least 25 s, which was sufficient to appropriately capture the dynamics of the underlying control process.

In our test paradigm, the vestibular input to postural control is not completely eliminated by having subjects in the supine orientation and closing their eyes to make them rely only on somatosensory information for performing the unipedal stance task. It is also possible that other motion cues, including rotation cues from the semicircular canals, and linear translation cues from the otolith organs, play a role in the performance of the posture task. In order to characterize and estimate the level of vestibular involvement in this test for each trial, we calculated the peak angular velocity in roll (Trv_p_), and the peak linear acceleration (Tay_p_) in the ML direction from the IMU placed approximately at the COM. To determine an estimate of peak linear acceleration in the ML direction at the level of the vestibular system (Hay_p_) located in the inner ear, we assumed a rigid body rotating about the ankle joint (this assumption was verified by high strategy scores: see “Results” and “Discussion” Sections), and multiplied Tay_p_ by a factor of 1.75 based on standard anthropometric dimensions (Winter, 2009). The peak angular velocity in roll experienced at the vestibular system (Hrv_p_) was the same as that at the COM (Trv_p_) because the whole body was assumed to be rotating rigidly around the ankle joint. Frequency analyses were carried out on Trv and Tay, to identify dominant frequencies during postural responses. Further, to quantify the relative time during the trials when vestibular information might have been available to be used for postural control in our test paradigm, the times spent by the subjects above the perceptual vestibular thresholds at the dominant frequencies for roll rotation and inter-aural translation as a percentage of the total trial duration were calculated.

A preliminary analysis showed no learning effect across the three trials for either of the two conditions. Thus, average values for UST, Hrv_p_, Hay_p_, strategy scores and times spent above perceptual thresholds in roll and inter-aural translation across all three trials, were calculated for each condition and each subject for further analyses. All of these parameters, except UST, for the two test conditions were compared using paired *t*-tests with a significance level set at 0.05. Since UST is not normally distributed because of the upper maximum limit (45 s), non-parametric Wilcoxon Signed Ranked test was carried out. Further, to compare PASS (UST = 45 s) or FALL (UST < 45 s), we used a contingency table and Fischer’s exact test. Multivariate analysis of variance (MANOVA) was carried out across the EO and EC conditions for the six parameters (the short-term and long-term diffusion coefficients-D_s_ and D_l_, scaling exponents-H_s_ and H_l_, critical time interval-Δ*t_c_*, and critical mean square displacement-Δ$r_{\text{c}}^{2}$) from the SDA analysis. When significant differences were found, *post hoc* tests were carried out to identify which parameters are different across the two conditions. The significance level was adjusted after accounting for multiple comparisons using appropriate Bonferroni correction (α = 0.008) for the different comparisons. SPSS version 21 (SPSS Inc., Chicago, IL, USA) was used for statistical analysis.

## Results

A total of 42 trials were conducted for each condition (14 subjects × 3 trials). For the EO condition, one subject fell after 25 s in his last trial, so it was not repeated. For the EC condition, three subjects fell during one of their trials and two subjects fell during two of their trials after the 25 s duration that was set for trial success. Consequently, those trials were not repeated. For two subjects in the EC condition, however, a fall happened before 25 s, so those trials were repeated although these subjects fell on the repeated trials as well. Overall, for the EO condition, there were 41 complete trials and one partial trial (*n* = 42). For the EC condition, there were 33 complete trials and seven partial trials (*n* = 40). All partial trials were performed by five of the subjects. The average UST was 44.4 ± 0.5 s (mean ± standard error) for the EO condition and 42.3 ± 1.23 s for the EC condition. Wilcoxon signed rank test revealed that there was no statistical difference between the two conditions (*p* = 0.116) for UST. Table 1 shows a contingency table for the PASS/FALL data. Fischer’s exact test on PASS/FALL data revealed that there was significant difference in the two conditions, and subjects were more likely to FALL under EC condition (*p* = 0.0146). This indicated that the EC condition presented a greater challenge to postural control than EO conditions.

**Table 1:** **Contingency table for FALL/PASS data for unipedal stance time (UST) for the two conditions tested**.

	Pass	Fall	Total
Eyes-open (EO)	41	1	42
Eyes-closed (EC)	33	9	42
	74	10	84

**Figure 4:**
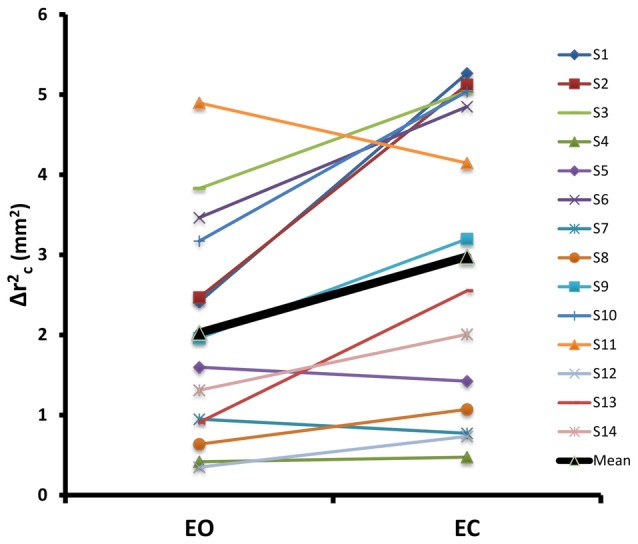
**Individual values and mean across all subjects for Δ$r_{\text{c}}^{2}$, for EO and EC conditions.** The two conditions were significantly different from each other for this parameter.

Table 2 shows means for the six SDA parameters for both EO and EC conditions along with the Romberg ratios (EC/EO: normalization of a parameter value during EC condition with the corresponding value during EO condition). Results of MANOVA revealed significant differences between EO and EC conditions across the six SDA parameters (Wilk’s Lambda = 0.224, *F*_(6,8)_ = 4.615, *p* = 0.026). *Post hoc* tests revealed that Δ$r_{\text{c}}^{2}$ was significantly different between the two conditions after accounting for Bonferroni correction (*F*_(1,13)_ = 10.94, *p* = 0.006). This indicated that a greater sway displacement occurred prior to the engagement of closed-loop control mechanisms during the EC conditions compared to the EO conditions. Figure 4 shows the individual values for the 14 subjects as well as the mean values across subjects for the two conditions for Δ$r_{\text{c}}^{2}$. Table 3 shows the Romberg ratios for the 14 subjects for Δ$r_{\text{c}}^{2}$ that ranged from 0.81 through 2.81 indicating the variability across subjects in sensory utilization for postural control.

**Table 2:** **Mean ± standard error of parameters from the stabilogram-diffusion analysis (SDA) for EO and EC conditions, along with the Romberg ratios**.

Stabilogram diffusion parameters	EO (*n* = 42)	EC (*n* = 40)	*p*-value	Romberg ratio
D_s_ (mm^2^/s)	2.28 ± 0.50	3.02 ± 0.61	0.071	1.52 ± 0.22
D_l_ (mm^2^/s)	0.29 ± 0.06	0.36 ± 0.06	0.194	1.79 ± 0.36
H_s_	0.413 ± 0.016	0.422 ± 0.013	0.382	1.03 ± 0.03
H_l_	0.166 ± 0.031	0.161 ± 0.022	0.845	1.16 ± 0.21
Δ*t_c_* (s)	0.473 ± 0.057	0.509 ± 0.054	0.430	1.13 ± 0.07
Δ$r_{\text{c}}^{2}$ (mm^2^)	2.026 ± 0.375	2.978 ± 0.506	0.006^a^	1.57 ± 0.15

*^a^Significant difference (*p* < 0.008) between EO and EC conditions*.

The strategy score for the EO condition was 87.8 ± 1.7% and that for the EC condition was 80.6 ± 2.9%. The high strategy scores verify that our subjects predominantly employed ankle strategy during the performance of the task matching our assumption for using a rigid body rotating about the ankle joint for estimation of linear accelerations at the level of the vestibular system. The estimated peak angular velocity (Hrv_p_) at the ear level was 1.3 ± 0.3°/s for the EO condition and 1.8 ± 0.4°/s for the EC condition. The estimated peak linear acceleration (Hay_p_) at the ear level was 0.049 ± 0.013 m/s^2^ for the EO condition and 0.070 ± 0.018 m/s^2^ for the EC condition. The dominant frequency across both conditions was found to be 0.26 ± 0.01 Hz for Hrv, and 0.61 ± 0.07 Hz for Hay. The time spent above the perceptual vestibular threshold for roll rotational velocity (2°/s at 0.26 Hz; Kolev, 2015) as a percentage of total trial duration was 0.44 ± 0.40% (0.20 ± 0.18 s) for the EO condition and 0.63 ± 0.32% (0.28 ± 0.14 s) for the EC condition. The time spent above the perceptual vestibular threshold for inter-aural translation (0.02 m/s^2^ at 0.61 Hz; Soyka et al., 2011; Valko et al., 2012) as a percentage of total trial duration was 8.4 ± 2.5% (3.8 ± 1.1 s) for the EO condition and 14.0 ± 4.0% (6.3 ± 1.8 s) for the EC condition. These results indicate that vestibular input to the postural control system was minimal both in magnitude and duration during the performance of each trial.

## Discussion

The current study assessed somatosensory utilization in a unipedal postural control task using the Gravity-Bed device in normal healthy adults. The results indicate that subjects relied primarily on somatosensory cues for postural control as relevant vestibular input was minimized during the performance of the postural stability task on the Gravity-Bed. Therefore, this study characterized and evaluated the unique contributions of somatosensory cues on postural equilibrium control.

Unipedal stance is the most challenging of balance tests in the Berg Balance Scale, which is a widely used and accepted clinical measure in functional balance testing (Berg et al., 1992). Results obtained using unipedal stance assessing lateral stability have greater ecological validity with respect to predicting the risk of falling (Maki et al., 1994) based on the rationale that falling predominantly occurs during phases of unipedal stance, as the base of support is smaller as compared to bipedal stance. All but two subjects were able to complete the unipedal balance trials in both conditions. The lack of difference between the USTs in the two conditions suggests that, overall fatigue (within a 45 s standing trial) was similar and likely small for the two conditions. However, our data suggest that the EC condition was more challenging for postural control since more falls occurred than during the EO condition. This is supported by previous studies (Collins and De Luca, 1995; Rougier and Farenc, 2000; Melzer and Oddsson, 2012) that have shown differences in EC and EO conditions using stabilogram-diffusion. In the Berg Balance Scale, the subject is required to stand unsupported on one-leg for at least 10 s to obtain the highest score in that test. However, to capture the spatio-temporal characteristics of the behavior adequately, we set the trial duration to 45 s on the basis of results from a study that investigated unipedal stance in EO and EC conditions in 549 normative subjects (Springer et al., 2007). Our pilot data suggested that subjects fatigued after six 45 s unipedal stance trials in our test paradigm, so we restricted our test to six trials in total (3 EO and 3 EC). If repetition was required, the maximal number of trials was limited to eight.

The ability to control posture with the unique contributions of all the somatosensory cues in the foot and ankle were captured by reducing the number of degrees of freedom needed to perform the task in our testing paradigm. Standing on one leg requires an initial voluntary action of moving the COM over the base of support of the forthcoming weight-bearing leg, followed by controlling the supported weight and maintaining alignment of different body segments (Jonsson et al., 2004). The trial timing was started only after the subject had completely stabilized and indicated that he/she was ready. This was similar to the *freeze* condition in Slobounov et al. (1997) in which the researchers instructed the subjects to restrict movements at all joints except the ankle joint allowing them to consider the motion of the whole body as an inverted pendulum.

Our finding of high strategy scores, close to 100 for both EO and EC conditions, indicate subjects mainly utilized an ankle strategy for postural control during the performance of the unipedal stance task in our test paradigm (Nashner and Peters, 1990; Neurocom, 2009; Vanicek et al., 2009). Additionally, hip movements are typically fast (1 Hz and above; Horak and Nashner, 1986; Neurocom, 2009), whereas sway movements about the ankle are slower (0.5 Hz or below), which is closer to what was observed in our frequency analyses. Overall, these results suggest that in our testing environment, subjects primarily followed directions and constraints imposed by the testing conditions and utilized an ankle strategy for controlling their posture. It should be further noted that in our test environment, subjects mainly relied on inversion or eversion at the ankle joint to maintain balance. In fact, ankle frontal plane proprioceptive sense has been shown to be an important predictor of UST (Allet et al., 2012) and also found to be significantly correlated with performance in elite athletes (Han et al., 2015).

Subjects used their dominant leg for testing in our study, and we do not believe results would have been different had they used their non-dominant leg (Fridén et al., 1989; Goldie et al., 1992; Tookuni et al., 2006; Zouita Ben Moussa et al., 2009; Kiyota and Fujiwara, 2014). Consequently, this type of testing paradigm may potentially be used for evaluation of unilateral injuries or disorders. Future investigations can be designed to use subjects as their own control for controlling the effects of body weight, anthropometry of the foot, anxiety, fatigue, concentration, experience and balancing strategies. It should be noted that since our testing was done only once, we cannot comment on the test-retest reliability of the parameters that we calculated.

SDA was conducted to gain insight into open- and closed-loop postural control behavior in our test paradigm. Previous studies have demonstrated that SDA parameters reflect changes in postural control associated with Parkinson’s disease (Mitchell et al., 1995), diabetic neuropathy (Toosizadeh et al., 2015), healthy aging (Collins et al., 1995), loss of foot sensation (Meyer et al., 2004), presence of vision (Rougier and Farenc, 2000; Melzer and Oddsson, 2012) and bipedal vs. unipedal stance (Hernandez et al., 2016). Our results showed that mean square critical displacement (Δ$r_{\text{c}}^{2}$) was significantly different between the two conditions. Higher values of Δ$r_{\text{c}}^{2}$ for the EC condition (Figure 4) are consistent with results from previous studies (Oddsson et al., 2007; Hernandez et al., 2016), indicating the average COP displacement at which the postural control process becomes predominantly anti-persistent (tendency to reverse direction) was higher during EC compared to EO condition. Consequently, in the EC condition when mainly somatosensory sensory cues were available, a greater sway displacement occurred prior to the engagement of closed-loop control mechanisms. Critical mean square displacement is a sensitive measure and has been shown to detect differences in children with attention deficit hyperactivity disorder (ADHD; Shorer et al., 2012) suggesting that voluntary perception would lead to lower critical displacement. Similarly, in our study, it is possible that during the EO condition, subjects were using visual cues from the environment and were better able to perceive changes in their body orientation. Lack of any difference in diffusion coefficients (D_l_ and D_s_) and scaling exponents (H_l_ and H_s_) across conditions suggest that the stochastic activity and degree of correlation between successive COP displacements were similar in both conditions as previously reported (Hernandez et al., 2016) for quiet unipedal upright stance in healthy adults.

It should be noted that the somatosensory index calculated from the sensory organization test (SOT) scores using the Neurocom Balance Manager (Natus Medical Incorporated, Pleasanton, CA, USA) is usually close to 1 (Neurocom, 2009; Dilda et al., 2014) with little variability across subjects. This lack of variability further advocates the need for a more sensitive test that can measure differences between subjects in their use of somatosensory information for postural control. In our test paradigm, the Romberg ratios for the parameters were more variable (Table 2) suggesting that the Romberg ratios can potentially be used to identify individuals who may have an inherent bias towards the use of somatosensory cues for postural control. The Romberg test is frequently used in posturography by comparing postural sway in EO and EC conditions. The ensuing Romberg ratio (EC/EO) is a set feature and it is interpreted as an indicator of somatosensory contribution to postural stability (Furman, 1994; MacDougall et al., 2006; Neurocom, 2009; Dilda et al., 2014; Moore et al., 2015). A ratio close to 1 implies that somatosensory information was sufficient to maintain balance even in the absence of vision. For example, the Romberg ratio for Δ$r_{\text{c}}^{2}$ for three of the subjects (see Table 3) was less than 1 suggesting that these subjects did not rely on visual input for balance control and were able to only use somatosensory cues to maintain balance in our test environment. This implies that they may have an inherent bias towards the use of somatosensory information. Romberg ratios for four of the subjects were between 1 and 1.4 (Table 3) suggesting that they were also able to adjust their strategy to rely on somatosensory cues while vision was occluded. The four subjects who had high Romberg ratios (>2) were more likely to be visually dependent as their balance was highly affected by the loss of visual information in the EC condition. The remaining three subjects with Romberg ratios between 1.4 and 2 were likely neither visually or somatosensory dependent.

**Table 3:** **Romberg ratio of each subject for critical mean square displacement (Δ$r_{\text{c}}^{2}$)**.

Sub. No.	Romberg Ratio
1	2.18
2	2.08
3	1.32
4	1.14
5	**0.89**
6	1.40
7	**0.81**
8	1.69
9	1.63
10	1.59
11	**0.85**
12	2.10
13	2.81
14	1.53

In order to quantify the levels of vestibular contribution (otoliths and semi-circular canals) that may have played a role in postural control in our test paradigm, the relevant characteristics of the motion exhibited by the subjects while performing this task were calculated and compared to known and previously reported thresholds that elicited perceptual and reflexive vestibular responses. The Hrv_p_ and Hay_p_ were of the same order of magnitude as the perceptual thresholds for roll rotation recognition by the vertical semi-circular canals and y-translation recognition by the utricle otolith organs, respectively (Benson et al., 1986; Soyka et al., 2011; Valko et al., 2012; Agrawal et al., 2013; Kolev, 2015). However, the average time spent above the perceptual threshold of roll-rotation was <1% of the total duration of the trial, and thus subjects would have had to rely on other cues for maintaining balance for the majority of the trial. It is also possible that the utricle otoliths received some transient translation cues, as the time spent above the perceptual threshold for inter-aural translation was ~8% for the EO condition and ~14% for the EC condition. However, it is unclear whether inter-aural translational cues are useful for eliciting postural corrections, especially in the supine orientation. It should be noted that the motion detection thresholds were obtained from studies that used standard oscillatory paradigms for direction recognition at specific frequencies. It is possible that the oscillatory nature of the stimulus in those studies made the stimulus predictable and thus resulted in a lower perceptual threshold. In our multi-frequency test environment, it may be that perceptual thresholds are higher. We infer from our results that in our supine testing environment, vestibular input available to the CNS for postural control is therefore minimized in magnitude and duration for which relevant information may be available during the performance of this task. Hence, in the absence of graviceptor tilt cues (both vestibular and non-vestibular) and minimized motion cues from the vestibular system, our testing environment emphasizes and necessitates an increased reliance on the somatosensory cues from the feet and ankle for postural control.

The Gravity-Bed can be used for implementation of functional rehabilitation exercises like squats, stepping and standing in clinical population who are unable to perform exercises in full weight bearing conditions such as in acute stroke survivors (Oddsson et al., 2015). More importantly, our results support the hypothesis that the improvement in upright postural control seen after exposing healthy subjects to resistance and balance training in a Gravity-Bed type environment must be related to enhanced use of somatosensory and/or visual information during training (Zemková and Oddsson, 2016). This has important implications for implementing countermeasure-training strategies for the spaceflight environment. The somatosensory system is a viable countermeasure target given its role in posture and locomotion control. Consequently, a well-optimized proprioceptive countermeasure coupled with preflight sensorimotor adaptability training (Bloomberg et al., 2015) may be required to sufficiently enable crewmembers to perform critical mission tasks after landing on a planetary surface. The Russian space program has utilized body axial loading systems (Penguin Suit) to minimize the effects of gravitational unloading to load the musculoskeletal system to activate the proprioceptive and body load sensing systems and preserve postural and locomotor function (Kozlovskaya and Grigoriev, 2004). Several studies have shown that dynamic foot stimulation studied during spaceflight missions, on the Salyut-6 and MIR space station as well as simulated microgravity conditions using the dry immersion and 60 days bed rest, restores absent neuromuscular activation during spaceflight throughout the entire lower-limb musculature (reviewed by Layne et al., 1998; Kozlovskaya et al., 2007; Layne and Forth, 2008; Reschke et al., 2009). Further, the information regarding reliance on somatosensory information can be used to test the efficacy of individualized training protocols designed to enable astronaut’s readapt more rapidly after spaceflight.

Baseline inter-trial variability of eye movement fluctuations measured during a simple predictive eye movement task have shown strong correlations with adaptability in a saccadic oculomotor task (Wong and Shelhamer, 2014). Previous studies have shown that subjects who rely more on vision for control of movement have more difficulty adapting their walking and postural control strategies in new sensorimotor environments, indicating that visual dependency may predict decreased ability to adapt to novel environments (Brady et al., 2009, 2012; Hodgson et al., 2010; Eikema et al., 2013). Similarly, inter-individual variability in somatosensory utilization may help explain variability in sensorimotor deficits and adaptability reported after spaceflight (Mulavara et al., 2010). This information regarding an individual astronauts’ reliance on somatosensory information can also be used to design efficacious individualized training protocols to enable astronaut’s readapt more rapidly after spaceflight (Seidler et al., 2015).

## Conclusion

Our results suggest that testing unipedal stance in the supine orientation environment of the Gravity-Bed can help assess and discriminate the use of information from the somatosensory receptors in the foot and ankle for postural control without imposing any limitations related to an intervention (e.g., anesthesia, vibration). Furthermore, the vestibular contribution even in a challenging postural control task during single limb stance is minimized allowing the utilization of somatosensory contributions in the Gravity-Bed environment. This is relevant for rehabilitation of patients who need to improve upright postural control but are unable to support full body weight bearing, including individuals with stroke (Oddsson et al., 2015) and more importantly for the training of astronauts (Bloomberg et al., 2015). The assessment of somatosensory contribution to postural control is important for populations such as the elderly (Riva et al., 2013), people with peripheral neuropathy (Rinalduzzi et al., 2016). In general, individual variability in utilizing sensory information affects adaptation to novel sensorimotor environments and can be used for designing personalized training protocols (Seidler et al., 2015).

## Author Contributions

RG helped design the protocol, analyzed the data and drafted the manuscript. YED and NEG performed data collection. EEC and BTP helped design the protocol. MFR and JJB helped design the protocol and with the interpretation of results. LIEO designed the Gravity-Bed, helped design the protocol, interpretation of results and review of the manuscript. APM conceived and designed the protocol, data analysis and helped in interpretation of results and drafting the manuscript.

## Funding

This study was supported in part by a grant from the National Space Biomedical Research Institute through NASA NCC9-58 to APM (PI, SA03801) and JJB (PI, SA02801) and by a grant from National Institute of Health to LIEO (PI, 5R21HD050655-02).

## Conflict of Interest Statement

LIEO is the inventor and owner of the patent for the Gravity-Bed device. The other authors declare that the research was conducted in the absence of any commercial or financial relationships that could be construed as a potential conflict of interest. The reviewer VR and handling Editor declared their shared affiliation, and the handling Editor states that the process nevertheless met the standards of a fair and objective review.
